# Of Mice and Men: The Inter-individual Variability of the Brain's Response to Drugs

**DOI:** 10.1523/ENEURO.0518-23.2024

**Published:** 2024-02-09

**Authors:** Wolfgang Löscher

**Affiliations:** Translational Neuropharmacology Lab, NIFE, Department of Experimental Otology of the ENT Clinics, Hannover Medical School, Hannover 30625, Germany

**Keywords:** antiseizure drugs, behavior, drug resistance, epigenetic changes, epilepsy, personalized medicine, psychopathology, rats, stress

## Abstract

Biological variation is ubiquitous in nature. Despite highly standardized breeding and husbandry under controlled environmental conditions, phenotypic diversity exists in laboratory mice and rats just as it does in humans. The resulting inter-individual variability affects various characteristics of animal disease models, including the responsiveness to drugs. Thus, the common practice of averaging data within an experimental group can lead to misinterpretations in neuroscience and other research fields. In this commentary, the impact of inter-individual variation in drug responsiveness is illustrated by examples from the testing of antiseizure medications in rodent temporal lobe epilepsy models. Individual mice and rats rendered epileptic by treatment according to standardized protocols fall into groups that either do or do not respond to antiseizure medications, thus mimicking the clinical situation in patients with epilepsy. Population responses are not normally distributed, and divergent responding is concealed in averages subjected to parametric statistical tests. Genetic, epigenetic, and environmental factors are believed to contribute to inter-individual variation in drug response but the specific molecular and physiological causes are not well understood. Being aware of inter-individual variability in rodents allows an improved interpretation of both behavioral phenotypes and drug effects in a pharmacological experiment.

## Significance Statement

Phenotypic diversity among individuals not only exists in humans but also occurs in laboratory rodents despite derivation from controlled populations and husbanding under standardized environmental conditions. As shown as an example of phenotypic diversity in this commentary, in rodent chronic epilepsy models, a proportion of animals fail to respond to antiseizure medications as is the case in humans with epilepsy. This recognition provides hope that animal models can be used to identify the underlying mechanisms of pharmacoresistance, which is a necessary first step in designing treatments that are effective in the large number of individuals with drug-resistant epilepsy (DRE). Thus, being aware of inter-individual variability allows an improved interpretation of behavioral phenotypes and experimental results in a pharmacological experiment.

## Inter-individual Variability in the Pharmacodynamics and Pharmacokinetics of Drugs

There can be dramatic differences in how individuals in a population respond to a particular drug (Kantae et al., 2017; Lonergan et al., 2017). The following factors are recognized as contributors to inter-individual variability in drug response:

1. Genetic variations can influence pharmacokinetics (drug absorption, distribution, metabolism, and elimination) and pharmacodynamics (therapeutic efficacy and adverse effects), a field of research that is termed pharmacogenomics.
2. Age-related changes in organ function, such as liver and kidney function, can impact drug metabolism and elimination, leading to variations in efficacy and tolerability.
3. Gender differences and hormonal state can influence drug response.
4. Body weight and composition may affect the distribution and elimination of drugs.
5. Diseases can alter drug metabolism and host responsiveness.
6. The concurrent use of multiple drugs can lead to interactions that affect the pharmacokinetics and pharmacodynamics of each drug.
7. Lifestyle factors such as diet, smoking, and alcohol consumption (either acute or chronic) can influence drug absorption and metabolism.
8. Individual variations in stress levels, psychological state, drug history, expectancy, and placebo responses can influence perceived drug efficacy and adverse effects.
9. Variations in the genotype and phenotype of the disease can lead to variability in drug response.

Drug formulation, route of administration, dosage, frequency and timing of administration, and adherence, are, of course, crucial to the therapeutic response but the factors listed above also play important roles in determining whether a drug therapy is effective. The numerous factors that can all cumulatively influence inter-individual variability in drug efficacy and tolerability make it difficult to identify the true causes of observed differences in drug response and the correlations among them (Lonergan et al., 2017). Understanding and addressing the heterogeneity of treatment effects among patients is crucial for effective personalized medicine, where treatments are tailored to an individual's unique genotypic or phenotypic characteristics to optimize therapeutic outcomes while minimizing side effects (Kantae et al., 2017; Goetz and Schork, 2018). However, although it is often assumed that patients respond differently to the same medical intervention, that is, some patients respond very well, while others respond poorly or not at all, it is important to differentiate “true” heterogeneity of drug responses among different patients from random variability, for example, by random temporal fluctuation in the state of the patient (Senn, 2004; Lonergan et al., 2017).

Thus, while some patients always and others never respond to treatment, a third group of patients may vary randomly in their response to a drug, which is, for instance, illustrated by the wax-and-wane pattern with a remitting–relapsing course during treatment with novel antiseizure medications (ASMs) in some patients with previously drug-resistant partial epilepsy (Schmidt and Löscher, 2005). The mechanisms underlying this transient or intermittent reversal of drug resistance are not understood but may be related to temporal fluctuations in epilepsy severity and/or lifestyle factors. An example of this phenomenon was described by Shorvon and Sander (1986), who reported an intermittent pattern in which active epilepsy is interrupted by periods of remission in 22% percent of a group of 181 patients with chronic uncontrolled seizures attending a specialized hospital outpatient service.

Such biological variation of the phenotype differs fundamentally from random noise, also known as measurement error, which refers to unexplained variability in the data (Voelkl et al., 2020). It affects the variation but not the size of an experimental treatment effect. A priori consideration of these issues, together with standardization of phenotypes, can increase the efficiency of stratified (“personalized” or “precision”) medicine by administering more appropriate treatments to specific subgroups of patients (Lonergan et al., 2017).

## Inter-individual Variability in Drug Responses Also Occurs in Mice and Rats

**Figure 1.**
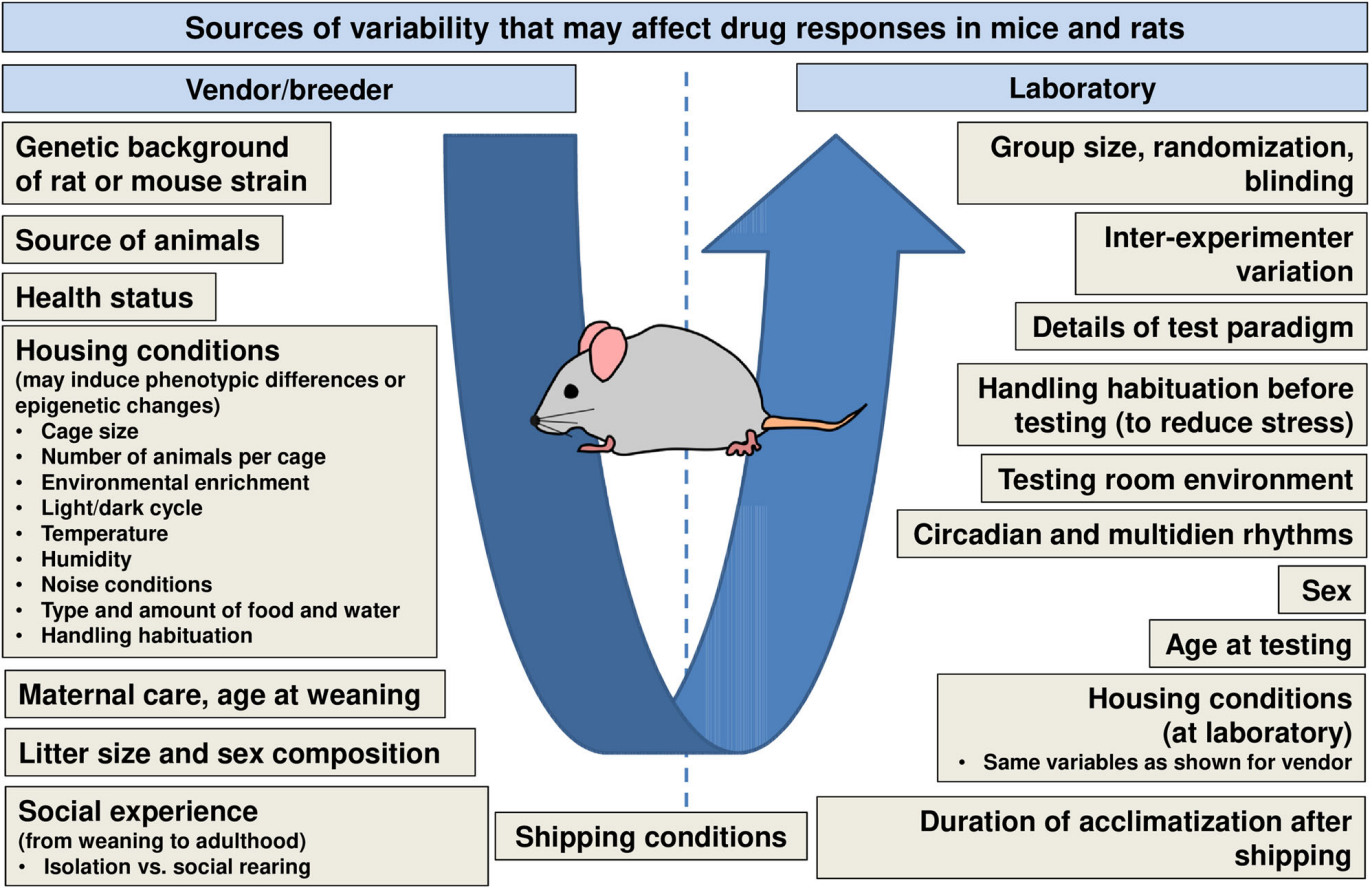
Sources of variability at animal vendors and in the laboratory that may affect the experimental outcome of drug studies in mice or rats. Several of the environmental variables shown may induce epigenetic changes. Modified from Löscher et al. (2017).

While inter-individual variability in drug response as a result of genotypic or phenotypic differences among patients is accepted in medical practice, it is often thought that such variability does not exist or is less impactful in research with laboratory rodents (Voelkl et al., 2020). Indeed, it is frequently assumed that the rigorous standardization in animal breeding and husbandry and the control of experimental conditions leads to more uniform drug responses. However, more recently, the existence of inter-individual variation in research with laboratory animals has been increasingly appreciated. There is now a general recognition that the disregard of biological variation in study design is a major cause of poor reproducibility in preclinical research involving animal models (Voelkl et al., 2020; van der Goot et al., 2021). Experimental results are recognized to vary not only with external environmental factors but also with the internal state of the animals as determined by genotype and differences acquired during development (Voelkl et al., 2020). As illustrated in Figure 1 and dealt with in detail previously (Johnson and Besselsen, 2002; Schellinck, 2010; National Research Council, 2011; Hanell and Marklund, 2014; Löscher et al., 2017), numerous variables, either at the animal vendor or in the laboratory performing the experiment, can affect drug responses in mice and rats. Not all of these variables can be easily controlled (or even appreciated at the time of scientific investigation). The same is true for inter-individual variability in drug response in mice and rats that occurs despite extensive environmental standardization and the use of genetically and microbiologically defined mice and rats of similar age and sex (van der Goot et al., 2021). As discussed below, inter-individual variability in rats and mice is not simply a matter of randomness but correlates with other phenotypic characteristics, indicating that there are true inter-individual differences in the disease state that are likely at play. Importantly, failure to recognize such variability may lead to the inappropriate interpretation of experimental results and poor reproducibility.

## Inter-individual Variability of the Brain’s Response to Drugs in Rodents

While inter-individual variability in drug responses of rodents may affect all fields of preclinical pharmacology, the body's most complex organ, the brain, is particularly susceptible. The examples chosen for this commentary relate to the medical treatment of epilepsy, the most common severe chronic neurological condition worldwide, which is characterized by spontaneous recurrent seizures (SRSs) and cognitive and behavioral comorbidities. About one-third of people with epilepsy have DRE in which chronic treatment with ASMs fails to control their seizures (Auvin et al., 2023). Mechanisms of DRE are only incompletely understood but in recent years various animal models of DRE have been created, and it is hoped that these models will provide insights into the mechanisms of pharmacoresistance and allow treatments that overcome drug nonresponding to be identified (Löscher et al., 2020; Löscher and White, 2023).

In the 1990s, the author's group first observed that outbred Wistar rats subjected to the amygdala kindling model of temporal lobe epilepsy (TLE) differ in their individual responses to ASMs (Löscher and Rundfeldt, 1991; Löscher et al., 1993). Upon repeated testing with the ASM phenytoin over several weeks, some kindled rats (∼20%) always responded with an increase in focal seizure threshold (responders), while other rats (∼20%) never responded (nonresponders), and a third group (∼60%) exhibited variable (or intermediate) responses; plasma levels of phenytoin were not significantly different in the three subgroups. We suggested that the three subgroups of amygdala-kindled rats model three different clinical scenarios (Löscher, 2006): (1) The responder subgroup models patients who achieve complete control of seizures during ASM treatment; (2) the nonresponder subgroup models drug-resistant patients with TLE in which ASM treatment does not significantly control or reduce seizure frequency; and (3) the variable responder group models patients in whom ASM treatment reduces seizure frequency but does not achieve complete control of seizures. We and numerous other groups replicated these inter-individual differences in the response to phenytoin by prospectively testing other large cohorts of kindled rats (Löscher, 2016; Löscher and White, 2023). Furthermore, kindled rats that were resistant to phenytoin were also resistant to several other ASMs and differed in several phenotypic and genetic aspects from phenytoin responders (Löscher and White, 2023), which argues against pure random variability in drug response (Senn, 2004). The inter-individual difference of kindled rats to phenytoin was selective for the effect of this ASM on kindled seizures, because the drug induced the same increase in the electroconvulsive threshold and the same extent of ataxia in both responders and nonresponders (Löscher and Rundfeldt, 1991).

Besides inter-individual genetic differences in outbred Wistar rats, another possible explanation for the different responses of individual kindled Wistar rats to ASMs would be the kindling process itself. Epileptic patients often initially respond to an ASM, but this effect may be lost with increasing duration of the disease, that is, when epilepsy becomes chronic (Schmidt and Löscher, 2005). To address the influence of kindling on the antiseizure response to phenytoin, Löscher's group tested phenytoin's antiseizure effect on the focal seizure threshold (via the amygdala electrode) before and after amygdala kindling in the same Wistar rats. Following kindling, rats were repeatedly tested with phenytoin to allow subgroup selection. Unexpectedly, in rats that were nonresponders after kindling, phenytoin exerted a significant antiseizure effect before kindling (Löscher et al., 2000). This study thus indicated that kindled phenytoin nonresponders become nonresponders through the kindling process, that is, the kindling-induced brain alterations. Because of the results of our breeding studies with kindled phenytoin responders and nonresponders (Ebert and Löscher, 1999), which indicated that the ability of kindled rats to respond or not to respond to phenytoin is, at least partially, genetically determined, the genetic background of an individual rat seems to determine whether it becomes a responder or nonresponder by kindling-induced limbic epileptogenesis (Löscher and White, 2023). We also checked whether the response to phenytoin after kindling was associated with any features of the Wistar rat population before they were kindled (e.g., weight, batch, season, sex, etc.) and did not find any such association (Ebert et al., 1999).

These findings in the kindling model led Löscher's group to hypothesize that—similar to human patients with epilepsy—epileptic rodents with spontaneous seizures also exhibit inter-individual variability in ASM responsiveness (Brandt et al., 2004). This hypothesis was evaluated by prospective experiments conducted in an outbred Sprague Dawley rat model in which the animals were rendered epileptic by sustained electrical stimulation of the basolateral amygdala (Brandt et al., 2003). Unlike the kindling model where seizures are elicited by amygdala stimulation, in this model the animals exhibit chronic spontaneous seizures with a broad range of daily rates, necessitating prolonged ASM administration for determining drug response. However, most ASMs are eliminated rapidly in rodents making it difficult to maintain effective blood levels during chronic treatment (Löscher and Klein, 2021). Phenobarbital (PB) is an exception with its long elimination half-life in rats of 9–20 h. PB was therefore chosen as the test ASM for these studies. Epileptic rats were continuously (24/7) monitored by video-EEG for SRS during a 2 week predrug control period, a subsequent 2 week period of chronic treatment with maximal tolerated doses of PB, and a 2 week postdrug monitoring period. Of note, the experimental conditions and investigative team were largely identical throughout the study, and animals were matched in age, sex, and environmental conditions.

**Figure 2.**
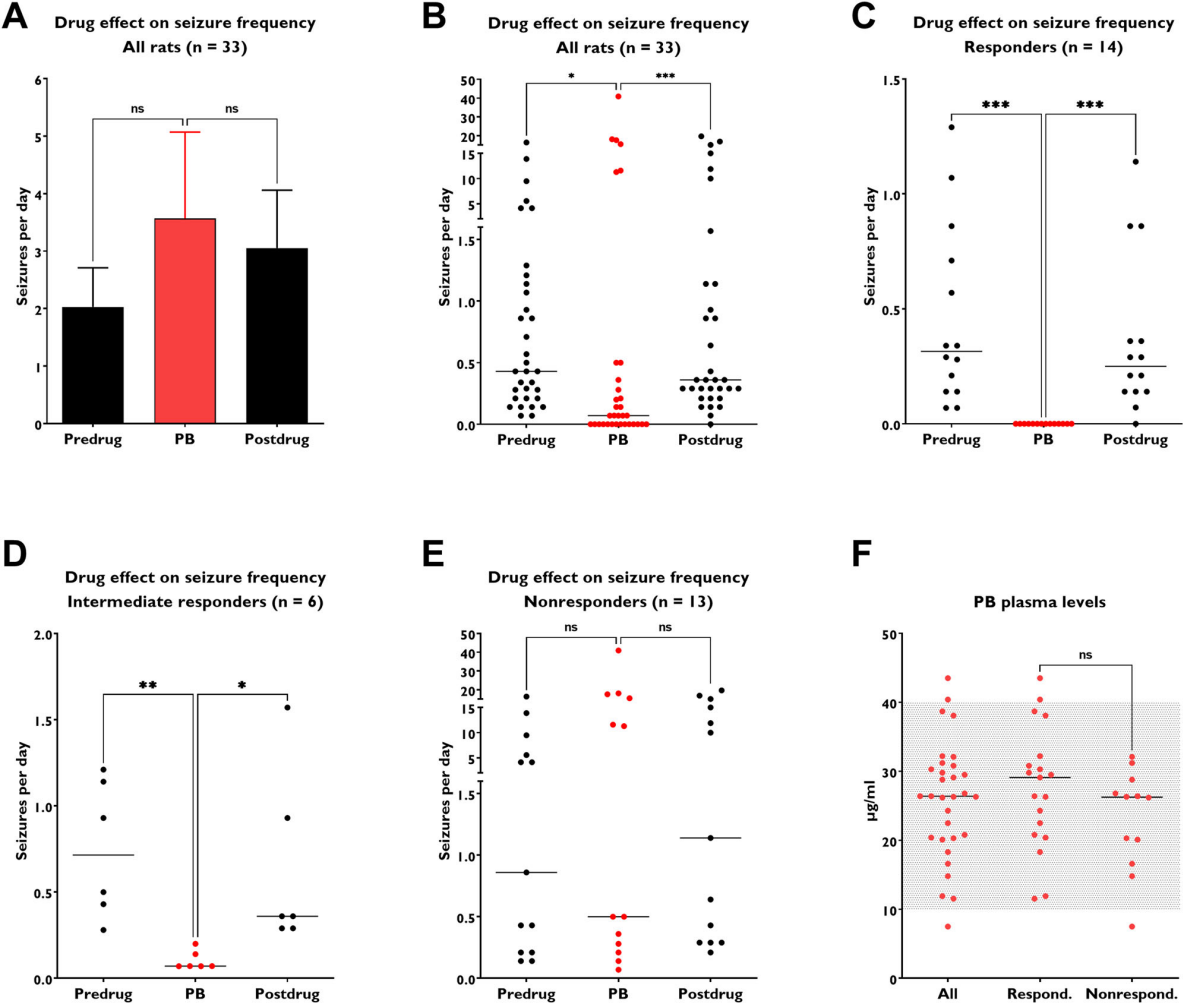
An example of inter-individual drug response in rats and how averaging data can lead to misinterpretation. In the experiment illustrated here, 33 epileptic Sprague Dawley rats with SRSs were treated with maximal tolerated doses of the ASM PB for 2 weeks. The effect of the treatment on the frequency of SRS was compared with SRS frequency in the 2 weeks before and after the treatment in the same rats. PB was administered twice daily at doses that led to the maintenance of therapeutic plasma levels (10–40 µg/ml) in all but one rat. In ***A***, all data were averaged (shown as means ± SEM) and statistically analyzed by one-way ANOVA for paired replicates, which indicated that PB did not significantly affect SRS frequency. However, both statistical normality tests (such as the Shapiro–Wilk test) and visual inspection of the individual data values in ***B*** indicate that SRS frequencies are not normally distributed but show clusters (or subgroups) of values in both the treatment and control periods with extremely high and very low SRS frequencies. With data of this sort, reporting the mean and SEM and the use of ANOVA may be misleading (Good and Hardin, 2012). As shown in ***B***, analysis of data by nonparametric ANOVA for paired replicates (Friedman test) and Dunn's multiple-comparisons test indicates that PB significantly suppressed SRS when compared with the predrug (*p < 0.05) and postdrug (***p < 0.001) control periods (control seizure frequencies did not differ between the two control periods). In the treatment period, partitioning of the data yielded three clusters (or subgroups): (1) rats in which PB prevents all seizures (responders); (2) rats in which PB decreases SRS frequency by at least 50% but the animals still exhibit SRS during treatment (intermediate responders); and (3) rats in which SRS frequency is not significantly affected by PB (nonresponders). In ***C–E***, the responder, intermediate responder, and nonresponder groups are shown, respectively. ***F***, The individual PB plasma levels determined during treatment. Each symbol represents the average trough level determined in several plasma samples per rat during the treatment period. The therapeutic plasma concentration reference range for PB (10–40 µg/ml) is indicated by the dotted area. Plasma drug levels in the responder/intermediate responder and nonresponder subgroups did not differ significantly. Data are from Löscher (2016) and were reanalyzed for this figure.

As shown in Figure 2*A*, the aggregate PB treatment appeared to cause no reduction in SRS frequency but tended to increase frequency. Comparisons among the groups using a conventional parametric statistical approach (one-way ANOVA) failed to demonstrate any significant differences in daily seizure frequency during the monitoring periods. However, inspection of the individual SRS frequency values for each animal clearly demonstrated that the animals fell into three groups, that is, responders, intermediate responders, and nonresponders (Fig. 2*B*), which was consistent with our predictions based on data obtained in the kindling model (see above). The statistical difference was revealed by a nonparametric statistical approach which was more appropriately applied because the frequency data are not normally (symmetrically) distributed. While the mean SRS value increased during PB treatment (Fig. 2*A*), the median value decreased substantially (from 0.43 to 0.07 seizures/day; *p* = 0.0195) since many of the animals did show a reduction in SRS frequency (Fig. 2*B*). Overall, ∼40% of the animals were protected from seizures by PB whereas 60% were not. The existence of several animals with very high seizure frequencies during PB treatment (seen in Fig. 2*B*) leads to a mean seizure frequency value numerically greater than the predrug or postdrug means (Fig. 2*A*). In clinical studies, it has been found that patients with high baseline seizure frequencies are more difficult to treat than those with low seizure frequency (Auvin et al., 2023), which has led to the “intrinsic severity hypothesis” of pharmacoresistance that posits that subjects presenting with high seizure rates have more severe forms of epilepsy and that the increased severity renders them more difficult to treat (Rogawski, 2013). Thus, for the data illustrated in Figure 2*B*, a subgroup analysis was performed to assess whether the well-recognized clinical observation regarding treatment responsiveness in individuals with low and high baseline seizure frequencies applies in the rat study. As shown in Figure 2*C*, 14/33 animals with low baseline seizure frequency (<1.5 seizures per day) exhibited no seizures during PB treatment, which was significant compared with both predrug and postdrug control periods. Thus the animals in this subgroup were “responders.” Another group of epileptic rats (6/33) with low baseline seizure frequency exhibited a significant (>50%) reduction in seizure frequency during PB treatment, but the rats still exhibited seizures and were thus considered “intermediate responders” (Fig. 2*D*). A third group of animals (13/33) with either low (*n* = 7) or high baseline seizure frequencies (>5 seizures per day) failed to show a significant reduction in SRS frequency during PB and were considered “nonresponders” (Fig. 2*E*). Thus, these data seem to indicate that the “intrinsic severity hypothesis” is not sufficient to explain drug resistance in this model, because 54% (7/13) of nonresponders had low seizure frequency (<1.5/day). We cannot exclude that state-dependent factors such as random temporal fluctuations in drug response (as discussed above for epilepsy patients) are involved in these findings, although the fact that the subgroup size (∼30–40%) of the PB nonresponders was replicated by us and others in subsequent studies in this model (see below) seems to argue against this possibility.

The striking difference in the response to PB in responders and nonresponders was not due to lower drug levels in the nonresponder group. All epileptic rats (except one) exhibited plasma drug levels within the clinically accepted therapeutic range of 10–40 µg/ml, and there was no significant difference between plasma levels in the responders and nonresponders (Fig. 2*F*). The findings in this study were replicated by prospective studies in our laboratory and also by other independent groups of researchers (Löscher, 2016; Löscher and White, 2023).

In subsequent experiments, we demonstrated that the segregation of animals into PB responder and nonresponder subgroups correlates with other phenotypic differences in addition to baseline seizure frequency. Nonresponders had greater hippocampal damage, more pronounced behavioral abnormalities, and higher brain expression of the multidrug transporter P-glycoprotein (Löscher, 2016; Löscher and White, 2023). Thus, similar to the inter-individual variability in drug responsiveness, there was also variability in the severity of epilepsy and its comorbidities, as is the situation in human patients with TLE (see detailed discussion in Löscher et al., 2020). Furthermore, as is also the case in humans with DRE, the resistance to PB in the rat model in the nonresponders extended to other ASMs (Löscher, 2016). While we do not fully understand the biological bases for the various differences between responders and nonresponders, genetic variability in the outbred animals as well as environmental factors are likely responsible (Ebert and Löscher, 1999; Voelkl et al., 2020).

Interestingly, we also found inter-individual variability in the response to ASMs in the inbred FVB/N mouse strain (Klein et al., 2015). In this study, animals were subjected to intrahippocampal injections of kainate, which led to highly frequent electrographic SRS. The animals were then tested for their response to six different ASMs. Similar to our findings in the outbred Sprague Dawley rats described above, the inbred mice also exhibited variation in responsiveness to the ASMs. Most nonresponders were resistant to more than one ASM. While inter-individual variability in a drug's response may be greater in outbred animals, it also can occur in an inbred strain. Such inbred animals have been considered to be genetically identical but recent studies have found that they are not isogenic (Chebib et al., 2021). Therefore, minor genetic differences may be sufficient to account for inter-individual differences in ASM responsiveness. Environmental factors (Fig. 1) could also contribute or may play a major role.

## Averaging Data Can Lead to Misinterpretations in Neuroscience

The examples of variation in ASM responsiveness described above are representative of observations in an increasing number of laboratory studies in neuroscience and other disciplines that have found wide variability in outcomes (Good and Hardin, 2012; Bernard, 2023; Marder, 2023). Laboratory experiments in experimental psychiatry are particularly prone to this phenomenon (Pitychoutis et al., 2011; Armario and Nadal, 2013; Aiello et al., 2015; Dellu-Hagedorn et al., 2018; Neyazi et al., 2018; van der Goot et al., 2021; Faure et al., 2022). As in our experience, it has often been noted that the common practice of averaging data can lead to erroneous conclusions.

Powering a preclinical study to account for potential inter-individual variability will require a larger number of animals, which is similar when—for instance—considering sex as a biological variable (Shansky, 2019). The increase in cost and time is commonly used as an objection. However, as illustrated by the example of inter-individual differences in the response of amygdala-kindled rats to the ASM phenytoin, potential trends of such variation are observed in relatively small groups of animals (Rundfeldt et al., 1990). Decisions can then be made whether to follow up with a larger prospective study explicitly designed to detect inter-individual variation in drug response as we did with the kindling model (Löscher and Rundfeldt, 1991) and, subsequently, with the epileptic rat model illustrated in Figure 2.

## Insights from Stress-Induced Models of Depression in Rodents

Recent studies have emphasized the importance of interactions between genetic factors and exposure to stressors in promoting psychiatric disorders (Radosavljevic et al., 2023). In psychiatry, stress, which can induce epigenetic changes, is believed to be a major environmental factor in disease variability. Chronic mild stress in rats is a widely used model to identify treatments for major depressive disorder. This model is characterized by striking inter-individual variability (Neyazi et al., 2018; Strekalova et al., 2022). It is noteworthy that stress may also play a role in inter-individual variability in epilepsy (Jhaveri et al., 2023). As in our study in the kindling model of DRE (Ebert and Löscher, 1999), selective breeding has been used to examine the potential mechanisms of inter-individual variability in models of psychiatric disorders (Allen et al., 2022).

## Variation in Disease Susceptibility

Even before inducing psychopathology or neurological disease, rodents of the same strain, sex, and age may differ in their intrinsic behavioral characteristics, which may affect the subsequent development of experimentally induced pathologies (Armario and Nadal, 2013; Theilmann et al., 2016; Faure et al., 2022; Strekalova et al., 2022; Löscher and White, 2023). For instance, inter-individual differences in baseline anxiety-like behavior (ALB) in Wistar rats affected subsequent amygdala kindling in that only rats with increased baseline ALB before kindling exhibited a lasting increase in ALB after kindling (Adamec and Shallow, 2000). Similar observations have been reported for various other behavioral phenotypes (Pitychoutis et al., 2011; Armario and Nadal, 2013; Freund et al., 2013; Theilmann et al., 2016; Dellu-Hagedorn et al., 2018; Allen et al., 2022). Thus, as in humans, the development of pathologies in laboratory rodents is related to individual differences determining either vulnerability or resilience. In line with this idea, numerous studies have reported marked inter-individual variability in the response of a laboratory rodent to stress that is suggestive of the presence of susceptible and resilient phenotypes (Strekalova et al., 2022). Similarly, individual differences of young rats in social play behavior, which is thought to be important for the development of brain and behavior, predict the sensitivity to self-administration of substances of abuse, including alcohol, later in life (Lesscher et al., 2021). A priori identification of phenotypic subgroups within an experimental pool enables researchers to actively control for any inter-individual variability in the design of their experiments (van der Goot et al., 2021). Incorporating inter-individual variability in experimental design and statistical analyses will likely improve the quality of results of many types of animal experiments and may avoid misinterpretation of pharmacological data.

## Conclusions

Inter-individual heterogeneity in drug responsiveness, affecting both drug efficacy and toxicity, is a major clinical problem in the pharmacotherapy of brain diseases such as epilepsy, depression, schizophrenia, bipolar disorder, and others (Butler, 2018; Stern et al., 2019; Löscher et al., 2020). The variation in drug responsiveness is caused by the interplay between drug pharmacology and the patients’ individual genetic and epigenetic background, developmental and environmental factors, and pathophysiological state. Given the potential existence of such variability, care should be taken when interpreting averaged data as the averages may hide subgroups that exhibit markedly different responsiveness. Although challenging, it will ultimately be necessary to develop a detailed understanding of the mechanistic bases of inter-individual variability if there is any hope of envisaging rational treatment strategies to overcome the drug resistance that occurs so frequently in epilepsy and other brain disorders.

Importantly, as shown by the examples of the animal models of DRE illustrated here, one can take advantage of inter-animal variability in drug responses because subgroups of responders and nonresponders can be ideally used to study mechanisms of drug resistance as done by my group and numerous other groups for epilepsy models in the past (Löscher and White, 2023). Enhanced understanding of these mechanisms may guide improved treatment and likely facilitate the development of more effective ASMs.

Overall, measures that might be taken to reduce or take advantage of inter-animal heterogeneity include (1) improved understanding of the parameters that influence an animal disease model; (2) ways to minimize or control for stress; and (3) consideration of the various other variables discussed in this commentary (and illustrated in Fig. 1).

However, as recently recommended for biomedical research using animal models (Voelkl et al., 2020), the use of systematic heterogenization of animal subjects by actively incorporating biological variation into study design will add more richly to the knowledge base than rigorous standardization that inevitably reduces the inference space. Furthermore, better understanding the sources of variability among animal responses in a single experiment, despite care with animal husbandry and attempts to standardize protocols within a lab, is likely to help improve the reproducibility of animal research or—alternatively—appreciate the unavoidable differences that resist standardization and render replicate studies so difficult.

## Synthesis

Reviewing Editor: Harald Sontheimer, University of Virginia Decisions are customarily a result of the Reviewing Editor and the peer reviewers coming together and discussing their recommendations until a consensus is reached. When revisions are invited, a fact-based synthesis statement explaining their decision and outlining what is needed to prepare a revision will be listed below. The following reviewer(s) agreed to reveal their identity: Raymond Dingledine.

Both reviewers are very complementary in their reviews and only suggest editorial changes. For example,

Reviewer 1 suggests several issues that need more clarification for example around "true heterogeneity of drug responses versus random variability"

Reviewer 2 suggest that the ideas put forth be collected together in a box or a bulleted list, perhaps in the "conclusions" section.

These all should be easy fixes
